# Supplementary figures and images for: The heparin-binding proteome in normal pancreas and murine experimental acute pancreatitis
Source: PLoS One. 2019 Jun 18;14(6):e0217633. doi: 10.1371/journal.pone.0217633 (PMC6581253; doi:10.1371/journal.pone.0217633)

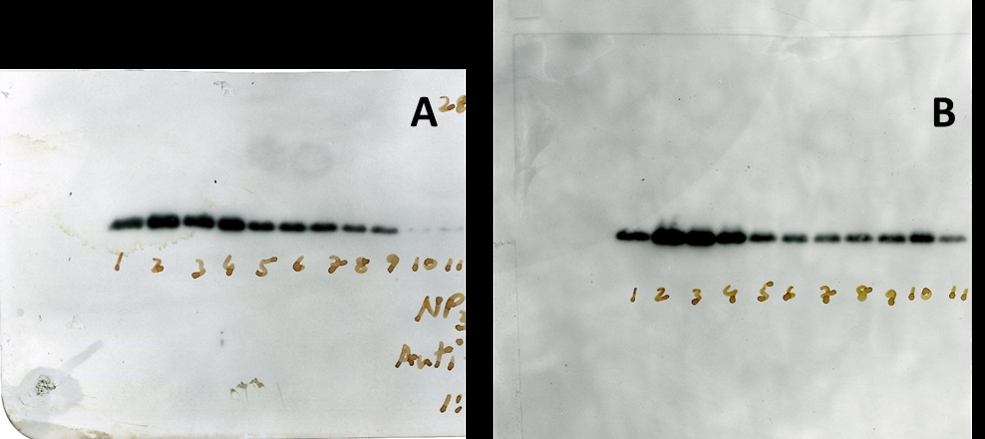

Supplement: S1 Fig — The enrichment of plasma membrane was assessed by western blot using an antibody against caveolin-1, which is a specific plasma membrane marker in (A) NP and (B) AP. (TIF) [file pone.0217633.s001.tif]

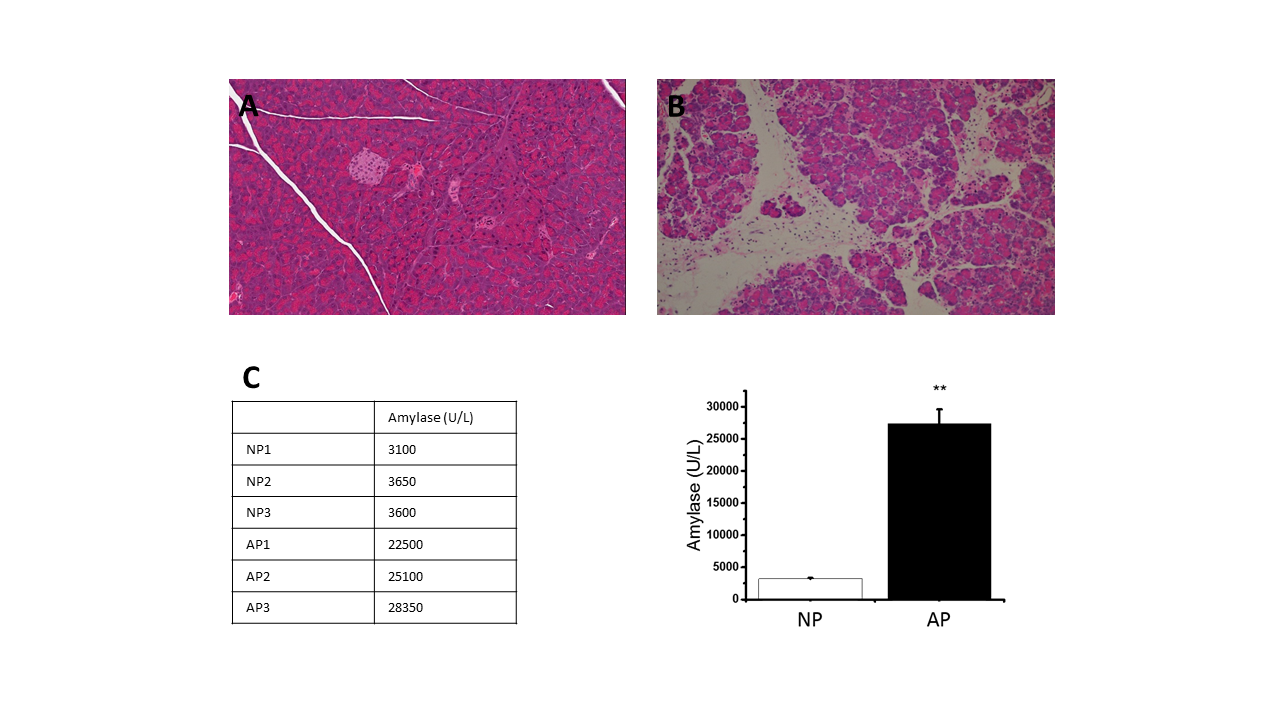

Supplement: S2 Fig — Representative images of H&E stained histology slides of A) NP with intact pancreas architecture and B) AP showing marked oedema, inflammatory cell infiltration and acinar cell necrosis. Mean serum amylase levels in (C) NP and AP. (TIF) [file pone.0217633.s002.tif]

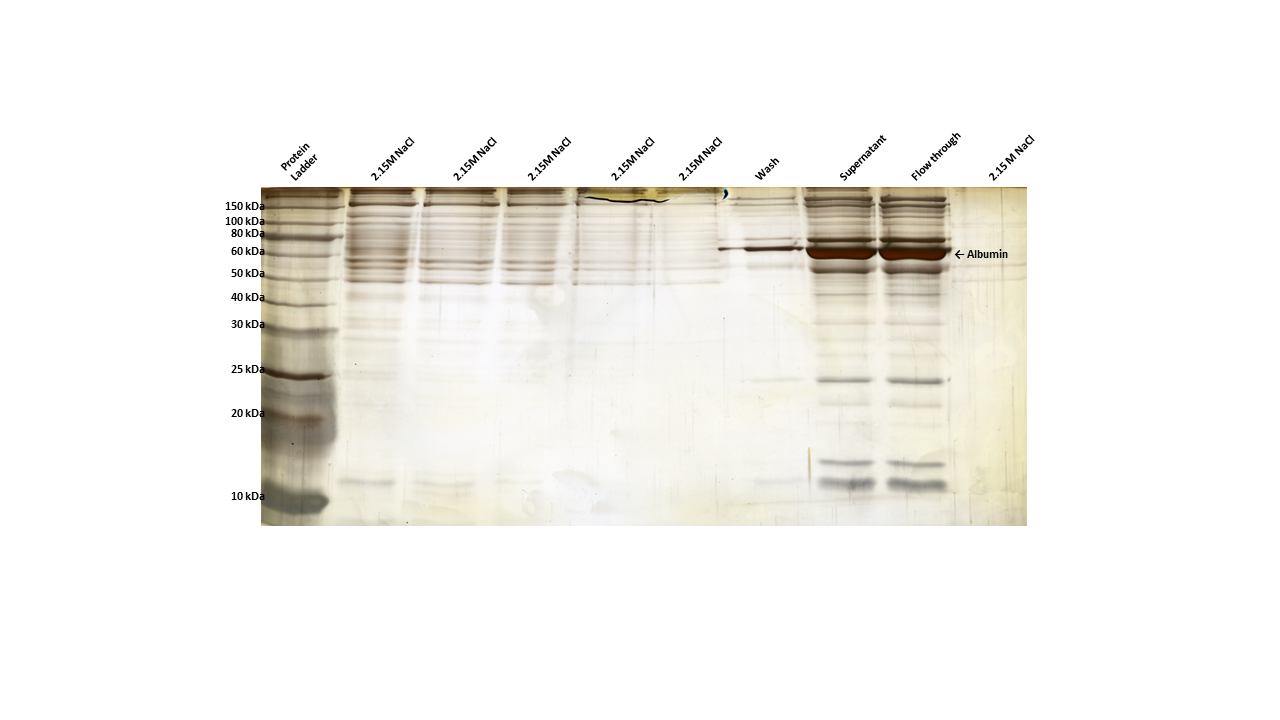

Supplement: S3 Fig — Murine plasma was diluted in PBS (1:8), and loaded onto a heparin column. The column was washed with PBS and further eluted using 2.15 M NaCl. Supernatant pre-loading onto the heparin column and the flow through were also examined and revealed that albumin levels decreased to negligible levels in the sample, which made effective MS analysis feasible. (TIF) [file pone.0217633.s003.tif]

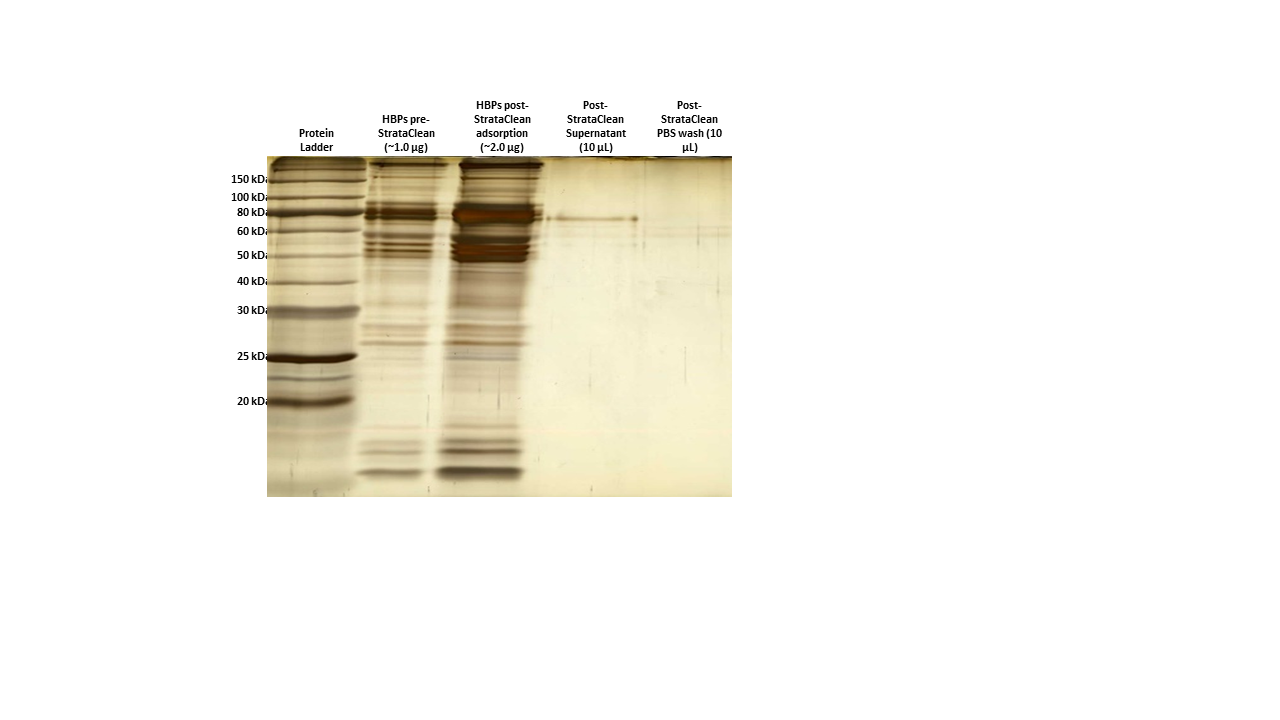

Supplement: S4 Fig — Following elution off the heparin column using 2.15 M NaCl (lane HBPs pre-StrataClean), ~100 μg was adsorbed onto 30 μL of StrataClean (Agilent Technologies). The supernatant (lane post-StrataClean supernatant) was removed after vortexing for 2 min and centrifugation at 2,000 × g. StrataClean resin was then washed with PBS and spun down (lane post-StrataClean PBS wash). The pellet was finally resupended in 170 μL H2O, 150 μL for mass spectrometry (MS), and the rest (approx. 20 μL) was resuspended with 10 μL of 2 × Laemmlli buffer and boiled for 5 min (lane HBPs post-StrataClean). Samples were loaded onto 12% SDS-PAGE and stained using silver staining. (TIF) [file pone.0217633.s004.tif]
